# Adverse Events Related to Azathioprine Use in Patients With Inflammatory Bowel Disease: A Real‐World Cohort Study

**DOI:** 10.1155/grp/1808791

**Published:** 2026-04-10

**Authors:** Mukesh Kumar Ranjan, Pradeep Neupane, Bigyan Maharjan, Manoj Lamsal, Bikash Poudel

**Affiliations:** ^1^ Department of Gastroenterology, Chitwan Medical College, Bharatpur, Chitwan, Nepal, cmc.edu.np; ^2^ Department of Gastroenterology, Tribhuvan University Teaching Hospital, Kathmandu, Nepal, teachinghospital.org.np

**Keywords:** adverse events, azathioprine, Crohn′s disease, IBD, leukopenia, thiopurines, tolerability, ulcerative colitis

## Abstract

**Background and Aim:**

Azathioprine is one of the commonly used maintenance therapies in patients with inflammatory bowel disease (IBD), specifically in low‐ and middle‐income countries. However, the use of thiopurines is questioned due to safety concerns. We aimed to assess the adverse event (AE) profile of azathioprine in IBD patients.

**Methods:**

This was a single‐centre retrospective study. All the consecutive patients treated with azathioprine were considered for this study. Data were collected from prospectively maintained IBD files. The primary objective was to assess the adverse events arising due to azathioprine use. The AEs were defined as per standard definitions. The relation between the AE and dose and duration of azathioprine use was assessed.

**Results:**

Among 48 patients [UC: 20 (41.7%) and CD: 28 (58.3%)] included, 30 (62.5%) were male. The mean age and the disease duration were 41.2 ± 15.7 years and 15 (5–40.5) months, respectively. The initiation and maximum dose of azathioprine were 0.91 ± 0.15 and 2.04 ± 0.58 mg/kg. The median thiopurine treatment duration was 6.5 (2.25–15), 11.5 (3.5–23.5) and 5.75 (2–12.5) months, respectively, in the whole cohort, UC and CD. A total of 25 (52.1%) patients developed adverse events [8 (40.0%) in UC and 17 (60.7%) in CD]. The commonest AEs were leukopenia in 15 (31.2%), GI intolerance in 5 (10.4%), arthralgia in 4 (8.3%), hepatitis in 3 (6.2%) and hair fall in 2 (4.1%) patients. No infection or acute pancreatitis episode or malignancy was reported. A total of 16 (33.3%) patients stopped azathioprine. AEs were the most common cause of azathioprine withdrawal in 12 (25.0%). No serious adverse event was reported.

**Conclusion:**

Adverse events are common and lead to therapy discontinuation in one fourth of the IBD patients on azathioprine. The commonest adverse events are leukopenia, GI intolerance, arthralgia, hepatitis, and hair fall.

## 1. Introduction

Inflammatory bowel disease (IBD) is a chronic intestinal disease characterised by episodes of remission and relapse. IBD, being a lifelong disease, significantly affects patients′ quality of life and psychological health [1, 2]. Moreover, every relapse or flare is associated with a significant financial burden [3]. This is why achieving and maintaining the state of remission is of utmost importance. Thiopurines have long been used as the maintenance therapy of choice. However, with the advent of newer targeted therapies, the treatment paradigm of IBD has seen a great shift from conventional therapies like thiopurines to advanced therapies (biologics and small molecules). Rightly so, because the advanced therapies offer a rapid onset of action and disease‐modifying property with an excellent efficacy profile. However, these newer drugs are expensive and not easily available. Azathioprine is an established pharmacotherapy for the maintenance of remission in patients with IBD. In resource‐limited settings, azathioprine continues to be an effective maintenance therapy of choice both for ulcerative colitis (UC) and Crohn′s disease (CD) [4]. In recent years, the use of azathioprine in IBD has been increasingly questioned largely because of the concerns related to adverse events. Adverse events occur in about one‐third of the patients on azathioprine [5]. About 40%–63% of the patients receiving thiopurines discontinue the drug, of which the commonest cause is adverse events, accounting for 10%–43% [6, 7]. The commonest adverse events associated with azathioprine are myelosuppression, gastrointestinal (GI) intolerance, hepatitis, acute pancreatitis, hair loss, and infections. The most feared adverse events are the development of malignancy, particularly lymphoma and nonmelanoma skin cancer, after long‐term use of azathioprine [8]. It is a well‐known fact that the adverse events, particularly myelosuppression, vary across different ethnic groups due to differential drug metabolism and genetic polymorphism [9, 10]. However, there is no data regarding the adverse events from this part of the world. Through this study, we aim to assess the adverse events associated with azathioprine use in Nepalese patients with IBD.

## 2. Methods

This was a single‐centre retrospective cohort study conducted at a tertiary referral hospital located in the central part of Nepal. The study was approved by the institute′s ethics committee (CMC‐IRC/082/083‐078), and recruited patients between January 2022 and September 2025. The data was extracted from IBD clinic files, where data is prospectively collected and entered manually at every patient visit. The data collected were patients′ demographic profiles, disease types and phenotypes, disease severity, extraintestinal manifestations, medications used, indications or use of azathioprine, dose and duration of azathioprine, adverse events and corresponding doses, duration of azathioprine, and cause of azathioprine discontinuation. We considered all the patients with IBD (both UC and CD) initiated on azathioprine for this study. Patients with incomplete details and the use of concomitant biologics were excluded. Since this is a retrospective study, written informed consent from the patients was waived. There was no TPMT and NUDT 15 gene polymorphism assessment done prior to starting azathioprine, as these were not available at our centre. We started azathioprine at a lower dose (usually 50 mg/day) and increased the dose by 25 mg every 2 weeks to reach a target dose of 2–2.5 mg/kg body weight daily. While doing so, we assessed for myelosuppression (complete blood count) and liver injury (liver function test) through blood investigations, and proactively obtained history for development of pancreatitis, hair fall, GI intolerance, arthralgia and myalgia at every visit. Once the patients were in clinical remission and free from adverse events, they were advised to follow up every 3–6 months. None of the patients received azathioprine and allopurinol combination. The primary objective of this study was to assess the adverse events due to azathioprine use and to evaluate the predictors of adverse events. The following criteria were used for the identification of adverse events [5, 11]:

• Myelosuppression was defined as the presence of white blood cell count <4 × 10^9^/L and/or neutrophils <1.5 × 10^9^/L and/or platelet count < 75,000/L. Mild, moderate and severe leukopenia were categorized as total leukocyte count 3–4 × 10^9^, 2–3 × 10^9^ and <2 × 10^9^/L, respectively. We considered a leukopenia cutoff as white blood cell count <4 × 10^9^/L because patients in our study were not assessed for TPMT and NUDT 15 gene polymorphism, leading to a heightened risk of leukopenia in these patients. South Asian patients with IBD treated with thiopurines are found to be more prone to develop leukopenia compared with their European counterparts [12]. The definition of leukopenia remains heterogeneous in the literature, with many studies considering a cut‐off of <4 × 10^9^/L [5, 13–15].
• Hepatotoxicity was defined as an increase in serum glutamic‐pyruvic transaminase (SGPT) or serum glutamic‐oxaloacetic transaminase (SGOT) greater than or equal to two times the baseline value in the absence of other obvious cause.
• Acute pancreatitis was defined as the presence of at least two of the findings among characteristic pancreatic pain, a rise in serum amylase and/or lipase by greater than or equal to three times the normal upper limit, and imaging suggestive of acute pancreatitis.
• GI intolerance was defined as symptoms such as nausea, vomiting, nonspecific abdominal pain in the absence of any other cause, and symptom onset after starting azathioprine, plus symptom resolution after stopping azathioprine.
• Hair fall: A significant increase in hair fall over the usual pattern in the absence of other explainable causes.
• Malignancy: Any malignancy, but notably the development of thiopurine‐associated malignancies like lymphoma, melanoma or nonmelanoma skin cancer, such as basal cell carcinoma.

### 2.1. Statistical Analysis

The data were analysed using STATA 14 software. Since patients with incomplete data were excluded, there were no missing data in the excel finalized for analysis. The categorical variables were expressed as numbers (%), and continuous variables as mean ± standard deviation or median (interquartile range) wherever applicable. To compare the adverse events between UC and CD, chi‐square test was used. For the assessment of predictors of adverse events, we performed univariate and multivariate analyses using logistic regression analysis. The parameters with a *p* value < 0.25 on univariate analysis were considered for multivariate analysis. Kaplan–Meier survival analysis was done for the relation between dose and the occurrence of adverse events. A two‐sided *p* value < 0.05 was considered significant.

Since this was retrospective study, we included all the eligible patients from the existing database.

## 3. Results

Among 182 patients screened for this study, a total of 48 IBD patients on azathioprine were included in the final analysis [UC: 20 (41.7%) and CD: 28 (58.3%)]. The mean age of the cohort was 41.2 ± 15.7 years, with males constituting 62.5% (*n* = 30). Age at disease onset was 38.5 ± 15.5, 38.6 ± 17.6 and 38.5 ± 14.2 years in the whole cohort, UC and CD, respectively. Of the 20 UC patients, 3 (15%), 11 (55%) and 6 (30%) patients had proctitis, left‐sided colitis and extensive colitis, respectively. Among the CD patients, 25 (89.3%) and 3 (10.7%) had inflammatory and stricturing disease, respectively. No patient had penetrating disease. Extraintestinal manifestations were present in 19 (39.6%) patients in the whole cohort (Table 1).

**Table 1:** Baseline characteristics.

	Total patients (*n* = 48)	UC (*n* = 20)	CD (*n* = 28)
Male, *n* (%)	30 (62.5)	13 (65.0)	17 (60.7)
Age, mean ± SD (years)	41.2 ± 15.7	42.8 ± 17.3	40.0 ± 14.7
Age at disease onset, mean ± SD (years)	38.5 ± 15.5	38.6 ± 17.6	38.5 ± 14.2
Body mass index, kg/m^2^	21.4 ± 3.1	21.2 ± 2.7	21.5 ± 3.4
Disease duration, median (IQR) (months)	15 (5–40.5)	32.5 (12–66)	12 (3–24)
Disease extent for UC			
E1 (proctitis), *n* (%)		3 (15)	
E2 (left‐sided colitis), *n* (%)		11 (55)	
E3 (extensive colitis), *n* (%)		6 (30)	
Crohn′s disease phenotype			
A1 (age < 17 years), *n* (%)			3 (10.7)
A2 (age 17–40 years), *n* (%)			13 (46.4)
A3 (age > 40 years), *n* (%)			12 (42.8)
B1 (nonstricturing, nonfistulizing), *n* (%)			25 (89.3)
B2 (stricturing), *n* (%)			3 (10.7)
B3 (fistulizing), *n* (%)			0
L1 (ileal disease), *n* (%)			16 (57.1)
L2 (colonic disease), *n* (%)			0
L3 (ileocolonic disease), *n* (%)			7 (25.0)
L4 (upper GI disease), *n* (%)			9 (32.1)
Perianal Crohn′s, *n* (%)			1 (3.5)
Comorbidities, *n* (%)	11 (22.9)	1 (5.0)	10 (35.7)
Hypertension, *n* (%)	3 (6.2)	0	3 (10.7)
Diabetes, *n* (%)	2 (4.1)	0	2 (7.1)
Hypothyroidism, *n* (%)	3 (6.2)	0	3 (10.7)
Anxiety, *n* (%)	4 (8.3)	1 (5.0)	3 (10.7)
Extraintestinal manifestations, *n* (%)	19 (39.6)	8 (40.0)	11 (39.3)
Aphthous ulcers, *n* (%)	11 (22.9)	4 (20)	7 (25.0)
Arthralgia (peripheral), *n* (%)	8 (16.6)	3 (15.0)	5 (17.8)
Ankylosing spondylosis, *n* (%)	3 (6.2)	1 (5.0)	3 (10.7)
Sacroiliitis, *n* (%)	3 (6.2)	2 (10.0)	1 (3.6)
Episcleritis/uveitis, *n* (%)	2 (4.1)	0	2 (7.1)
Use of other medications, *n* (%)			
5‐ASA derivatives	23 (47.9)	10 (50.0)	13 (46.4)
Corticosteroids	5 (10.4)	2 (10.0)	3 (10.7)

Abbreviations: 5‐ASA, 5‐aminosalicylic acid; CD, Crohn’s disease; UC, ulcerative colitis.

### 3.1. Indications of Azathioprine Use

The commonest indication for azathioprine initiation was steroid‐dependent disease in 22 (45.8%) patients, followed by maintenance therapy in CD in 21 (43.7%) patients. Since biologics are expensive and not readily available, and upadacitinib and injectable methotrexate are not available, azathioprine remains the maintenance therapy of choice in our CD patients. Other indications for azathioprine use were post acute severe ulcerative colitis (ASUC) use, steroid‐refractory disease and perianal CD in 3 (10.7%), 1 (2.1%) and 1 (2.1%) patient, respectively.

### 3.2. Thiopurine Use and Adverse Events

The dose of azathioprine initiation was 0.91 ± 0.15 mg/kg/day in the whole cohort (UC: 0.88 ± 0.16 mg/kg and CD: 0.93 ± 0.14 mg/kg). The maximum azathioprine dose reached was 2.04 ± 0.58 mg/kg (UC: 2.00 ± 0.54 mg/kg and CD: 2.07 ± 0.62 mg/kg). The median thiopurine treatment duration was 6.5 (2.25–15), 11.5 (3.5–23.5) and 5.75 (2–12.5) months, respectively, in the whole cohort, UC and CD.

A total of 25 (52.1%) patients on azathioprine developed adverse events. The commonest adverse events were myelosuppression in 15 (31.2%), GI intolerance in 5 (10.4%), arthralgia in 4 (8.3%), hepatitis in 3 (6.2%) and hair fall in 2 (4.1%) patients. Of the 15 patients with myelosuppression, 9 patients had mild and 6 patients had moderate leukopenia, whereas no patient developed severe leukopenia. The adverse events were statistically comparable between UC and CD (*p* = 0.15). No patient developed acute pancreatitis. No cases of lymphoma or skin malignancy were seen (Table 2).

**Table 2:** The adverse events of thiopurine use.

Adverse events	Whole Cohort (*n* = 48)	UC (*n* = 20)	CD (*n* = 28)	*p*value	Chi^2^ value
Any adverse event, *n* (%)	25 (52.1)	8 (40.0)	17 (60.7)	0.15	2.00
Myelosuppression/leukopenia, *n* (%)	15 (31.2)	6 (30.0)	9 (32.1)	0.87	0.02
Gastrointestinal intolerance, *n* (%)	5 (10.4)	0	5 (17.8)	0.05	3.98
Arthralgia, *n* (%)	4 (8.3)	1 (5.0)	3 (10.7)	0.44	0.49
Drug‐induced liver injury, *n* (%)	3 (6.2)	1 (5.0)	2 (7.1)	0.62	0.09
Hair fall, *n* (%)	2 (4.1)	1 (5.0)	1 (3.6)	0.66	0.05
Infections, *n* (%)	0	0	0		
Acute pancreatitis, *n* (%)	0	0	0		
Malignancies, *n* (%)	0	0	0		

*Note:* Four patients had overlapping adverse events.

**Figure 1:**
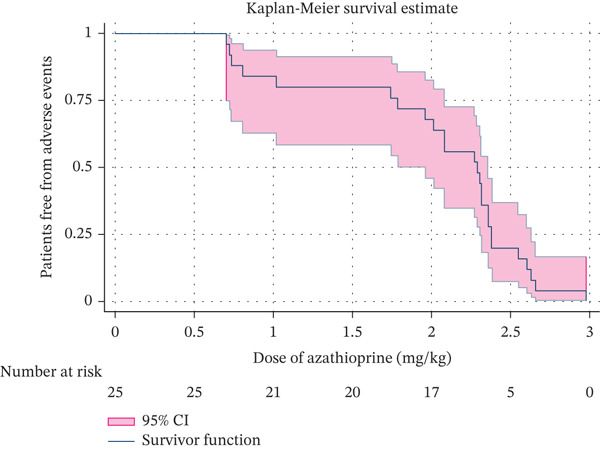
The Kaplan–Meier survival analysis shows a sharp decline in the number of patients free from adverse events after azathioprine dose increased beyond 2 mg/kg.

Myelosuppression occurred at a median dose and duration of 2.3 (2.0–2.5) mg/kg and 12 (2.5–14) months, respectively. Similarly, GI intolerance, arthralgia, hepatitis and hair fall occurred at median dose and duration of 0.8 (0.7–1.7) mg/kg and 0.5 (0.5–1.3) months, 1.54 (0.7–2.3) mg/kg and 1.5 (0.5–10.7) months, 2.3 (2.0–2.6) mg/kg and 12 (2.5–144) months and 2.3 (1.7–2.4) mg/kg and 7.2 (0.5–14) months, respectively (Table 3). The adverse events increased with increasing dose. There was a sharp increase in adverse events at an azathioprine dose > 2 mg/kg body weight (Figure 1).

**Table 3:** Adverse events and relationship with dose and duration of azathioprine.

Adverse events (AE)	Dose at the adverse events (mg/kg)	Duration of thiopurine use at AE (months)
Any adverse events	2.29 (1.78–2.38)	4 (2–13)
Myelosuppression/leukopenia	2.3 (2.0–2.5)	12 (2.5–14)
Gastrointestinal intolerance	0.8 (0.7–1.7)	0.5 (0.5–1.3)
Arthralgia	1.54 (0.7–2.3)	1.5 (0.5–10.7)
Drug‐induced liver injury	2.3 (2.0–2.6)	12 (2.5–144)
Hair fall	2.3 (1.7–2.4)	7.2 (0.5–14)

A total of 16 (11 in UC and 4 in CD) patients stopped thiopurine due to various causes, and the remaining 32 (66.6%) were still on azathioprine at the last follow‐up. The causes of azathioprine discontinuation were the development of adverse events in 12 (25.0%), failure of therapy in 3 (6.2%) and self‐discontinuation in 1 (2.1%) patient. After the occurrence of adverse events, azathioprine was stopped in 12 (25%) patients, dose reduction was done in 12, and switched to 6‐mercaptopurine in one patient. With these adjustments, all the adverse events, including leukopenia, resolved. GI intolerance, myelosuppression and hepatitis were the reasons for azathioprine discontinuation in five, four and three patients, respectively. No serious adverse events or mortality were reported.

### 3.3. Predictors of Adverse Events

For univariate analysis of predictors of adverse events, age, sex, body mass index, disease type, age at disease onset, any extraintestinal manifestation, any comorbidities, dose at azathioprine initiation, dose at adverse events, disease duration at azathioprine initiation, age at azathioprine initiation and duration of azathioprine use were used as independent variables. None of these parameters predicted adverse events. The variables with *p* value < 0.25 (disease type, any extraintestinal manifestation and disease duration at azathioprine initiation) were used for multivariable analysis. These parameters were not found to predict the development of adverse events on multivariate analysis (Table 4).

**Table 4:** Predictors of adverse events.

	Univariate analysis			Multivariate analysis		
Variables	OR (95% CI)	*Z*‐value	*p*value	OR (95% CI)	Z‐value	*p*value
Sex	0.8 (0.24–2.58)	−0.37	0.70			
Age	0.98 (0.94–1.02)	−0.87	0.38			
BMI	1.04 (0.86–1.25)	0.44	0.65			
Disease type	0.43 (0.13–1.39)	−1.41	0.16	0.50 (0.14–1.72)	−1.09	0.27
Age at disease onset	0.98 (0.95–1.02)	−0.61	0.54			
Any EIM	2.10 (0.64–6.90)	1.23	0.21	2.53 (0.69–9.16)	1.42	0.15
Any comorbidities	0.70 (0.18–2.73)	−0.50	0.61			
Dose at AZA initiation	0.72 (0.01–30.16)	−0.17	0.86			
Dose at AE	0.77 (0.28–2.07)	−0.51	0.61			
Disease duration at AZA initiation	0.98 (0.95–1.00)	−1.33	0.18	0.98 (0.95–1.00)	−1.31	0.19
Age at AZA initiation	0.98 (0.94–1.02)	−0.87	0.38			
Duration of azathioprine use	1.02 (0.97–1.06)	0.94	0.34			
Concomitant use of 5‐ASA	0.51 (0.16–1.61)	−1.14	0.25			
Concomitant use of corticosteroids	0.57 (0.08–3.82)	−0.57	0.57			

Abbreviations: 5‐ASA, 5‐aminosalicylic acid; AZA, azathioprine; BMI, body mass index; EIM, extraintestinal manifestation; OR, odd′s ratio.

## 4. Discussion

In this single‐centre retrospective cohort study, we found that 52.1% IBD patients who were started on azathioprine developed adverse events. The commonest adverse events were myelosuppression, GI intolerance, arthralgia, hepatitis and hair fall. One third of the patients on azathioprine discontinued the drug, of which the commonest cause was the development of adverse events, accounting for 25% of the whole cohort. We also found that these adverse events resolved with adjustments like discontinuing azathioprine, reducing the dose and switching to 6‐MP.

Azathioprine is an age‐old pharmacotherapy used effectively to maintain remission both in UC and CD [16]. However, with associated adverse events, its use in IBD has been questioned in recent years [17]. In the current study, more than half of the patients on azathioprine developed adverse events, which is significantly higher than the reported rates of 20%–33% from other Asian and Western centres [5, 18, 19]. This is a significant finding that indicates that Nepalese IBD patients on azathioprine are at heightened risk of adverse events. A retrospective cohort study from Spain also reported a higher (49%) adverse event rate, similar to the current study [20]. This could be due to the different patient population in our cohort. In addition, our cohort demonstrated a higher rate of myelosuppression (60% of all the adverse events), which has influenced the overall adverse events. Myelosuppression was seen in 15 (31.2%) of the whole cohort, which is higher than reported rates of 4%–12% from other centres across the world [18, 19, 21, 22]. The higher rate of myelosuppression in the current study could be because of the absence of TPMT and NUDT polymorphism assessment before starting azathioprine. Moreover, we considered myelosuppression as a total leukocyte count lower than 4 × 10^9^/L, whereas other studies considered myelosuppression as leukocyte counts <3 × 10^9^/L [18, 23]. Similar to our study, a retrospective study of a large database from North India that used similar criteria for myelosuppression reported a higher myelosuppression rate of 23.4% [5]. However, none of the patients developed severe leukopenia in our cohort. This finding might be largely because azathioprine was initiated at a lower dose and dose escalation was done based on complete blood count at each visit, which might have resulted in diagnosis of myelosuppression at an early stage. A meta‐analysis has also illustrated that the occurrence of severe leukopenia is rare in IBD patients treated with azathioprine [21]. The median time to myelosuppression in the current study was 12 (2.5–14) months and occurred at a median dose of 2.3 (2.0–2.5) mg/kg. This underscores the fact that myelosuppression is a dose‐dependent adverse event of azathioprine [16, 24, 25].

The other common adverse events were GI intolerance in 10.4%, arthralgia in 8.3%, hepatitis in 6.2% and hair fall in 4.1% patients. These adverse event rates are in line with studies from India but lower compared with those from other countries. This finding could primarily be because the cohort in our study resembles that of neighbouring country India, plus azathioprine was started at a lower dose and escalated gradually [5, 19]. Whereas, at other centres, azathioprine was started at 2–2.5 mg/kg, which might have resulted in higher rates of the above‐mentioned adverse events [20]. Of these adverse events, GI intolerance occurred at a lower median dose of 0.8 (0.7–1.7) mg/kg and a median duration of 0.5 (0.5–1.3) months, which further emphasises the idiosyncratic nature of these adverse events [11, 16]. Acute pancreatitis was not seen in the current cohort. This might be because acute pancreatitis is a relatively rare adverse event, and the sample size of this study was small [11]. No serious adverse event requiring hospitalization was reported. No case of malignancy was reported. However, it is important to acknowledge that the sample size was small, and the median follow‐up was short to reflect on the incidence of malignancy.

Despite half of the patients developing adverse events, only one‐fourth of the entire cohort discontinued azathioprine due to adverse events, which is in line with other studies done previously [26–28]. All the adverse events in the current study resolved after stopping azathioprine, reducing the ongoing dose and switching to 6‐MP. This demonstrates that these short‐term adverse events due to azathioprine use are nonserious and can be easily managed.

The data on predictors of adverse events are scarce and inhomogeneous. In a retrospective study conducted at a UK district hospital, female sex and age more than 50 years were the predictors of azathioprine intolerance [26]. In the current study, none of the parameters were found to be a predictive factor of adverse event occurrence on univariate and multivariate analysis. This could be because of a relatively small sample size and a smaller number of adverse events other than myelosuppression. However, an Indian study with a sample size of 1093 also failed to find predictors of adverse events [5].

There are certain limitations of this study that need to be acknowledged. This is a single‐centre study prone to limitations of retrospective data collection. Moreover, a small sample size might have also resulted in the absence of rare adverse events like pancreatitis. The median follow‐up duration of the cohort was short, making it unsuitable to address the adverse events like lymphoma and nonmelanoma skin cancers. We did not perform TPMT and NUDT15 polymorphism testing, which is recommended before initiating azathioprine [29]. This might have resulted in a relatively higher occurrence of myelosuppression in our cohort. These genetic polymorphism tests are not available at our centre and are expensive when outsourced. Due to a small sample size, the overall number of adverse events was also small, making this study unsuitable to evaluate the predictors of individual adverse events. Mild leukopenia is considered a predictor of thiopurine effectiveness in IBD patients. Though data is inconsistent in this regard, we did not evaluate the effectiveness of azathioprine in our study. Nevertheless, to the best of our knowledge, this is the first study from Nepal to assess this important aspect of azathioprine use. For a country like Nepal, where IBD is rising, and many aspects of this disease are still unexplored [30], we believe this study will help clinicians anticipate and deal with azathioprine‐related adverse events. The outcome of this study represents a unique population; hence, this may not be suitable for generalization.

## 5. Conclusion

This single‐centre study demonstrates that adverse events occur in more than half of the patients with IBD on azathioprine. However, only about one‐fourth of the patients discontinued azathioprine due to adverse events. The commonest adverse events were myelosuppression, GI intolerance, arthralgia, drug‐induced hepatitis and hair fall. All these adverse events were managed with adjustments like drug discontinuation, dose reduction or switching to 6‐MP. No serious adverse events were noted. However, studies with a large sample size and substantial follow‐up duration would be suitable to explore the incidence of malignancies and other rare adverse events.

## Funding

No funding was received for this manuscript.

## Conflicts of Interest

The authors declare no conflicts of interest.

## Data Availability

The data that support the findings of this study are available from the corresponding author upon reasonable request.

## References

[1] Mitropoulou M. A. , Fradelos E. C. , Lee K. Y. , Malli F. , Tsaras K. , Christodoulou N. G. , and Papathanasiou I. V. , Quality of Life in Patients With Inflammatory Bowel Disease: Importance of Psychological Symptoms, Cureus. (2022) 14, no. 8, e28502, 10.7759/cureus.28502, 36185946. 36185946 PMC9514670

[2] Singh A. , Bhardwaj A. , Tripathi A. , Ranjan M. K. , Singh D. , Sachdeva A. , Marwah M. , Sadana K. S. , Bansal N. , Mahajan R. , Kaur K. , Midha V. , and Sood A. , Burden of Anxiety, Depression and Perceived Stress in Patients With Inflammatory Bowel Disease: A Cohort Study From North India, Digestive Diseases and Sciences. (2024) 69, no. 3, 775–790, 10.1007/s10620-023-08242-3, 38282185. 38282185

[3] Yadav A. I. , Rana V. S. , Patil A. N. , Pathak P. , Dutta U. , and Sharma V. , Comparison of Cost of Care in Patients With Flares vs. Remission in Ulcerative Colitis: A Perspective From a Developing Country, Medicine International. (2025) 5, no. 6, 10.3892/mi.2025.274, 41159093. PMC12555847 41159093

[4] Ranjan M. K. , Kumar P. , Vuyyuru S. K. , Kante B. , Mundhra S. K. , Golla R. , Virmani S. , Sharma R. , Sahni P. , das P. , Kalaivani M. , Upadhyay A. D. , Makharia G. , Kedia S. , and Ahuja V. , Thiopurines Have Sustained Long-term Effectiveness in Patients With Inflammatory Bowel Disease, Which Is Independent of Disease Duration at Initiation: A Propensity Score Matched Analysis, Journal of Crohn′s and Colitis. (2024) 18, no. 2, 192–203, 10.1093/ecco-jcc/jjad135, 37584328. 37584328

[5] Ranjan M. K. , Kante B. , Vuyyuru S. K. , Kumar P. , Mundhra S. K. , Golla R. , Sharma R. , Sahni P. , das P. , Makharia G. , Kedia S. , and Ahuja V. , Minimal Risk of Lymphoma and Non-Melanoma Skin Cancer Despite Long-Term Use of Thiopurines in Patients With Inflammatory Bowel Disease: A Longitudinal Cohort Analysis From Northern India, Journal of Gastroenterology and Hepatology. (2022) 37, no. 8, 1544–1553, 10.1111/jgh.15880, 35501287. 35501287

[6] Macaluso F. S. , Renna S. , Maida M. , Dimarco M. , Sapienza C. , Affronti M. , Orlando E. , Rizzuto G. , Orlando R. , Ventimiglia M. , Cottone M. , and Orlando A. , Tolerability Profile of Thiopurines in Inflammatory Bowel Disease: A Prospective Experience, Scandinavian Journal of Gastroenterology. (2017) 52, no. 9, 981–987, 10.1080/00365521.2017.1333626, 2-s2.0-85019707497, 28554266. 28554266

[7] Fraser A. G. , Orchard T. R. , and Jewell D. P. , The Efficacy of Azathioprine for the Treatment of Inflammatory Bowel Disease: A 30 Year Review, Gut. (2002) 50, no. 4, 485–489, 10.1136/gut.50.4.485, 2-s2.0-0036278519, 11889067. 11889067 PMC1773162

[8] Gómez-García M. , Cabello-Tapia M. J. , Sánchez-Capilla A. D. , De Teresa-Galván J. , and Redondo-Cerezo E. , Thiopurines Related Malignancies in Inflammatory Bowel Disease: Local Experience in Granada, Spain, World Journal of Gastroenterology. (2013) 19, no. 30, 4877–4886, 10.3748/wjg.v19.i30.4877, 2-s2.0-84884518355, 23946592. 23946592 PMC3740417

[9] Dewit O. , Moreels T. , Baert F. , Peeters H. , Reenaers C. , de Vos M. , van Hootegem P. , Muls V. , Veereman G. , Mana F. , van Outryve M. , Holvoet J. , Naegels S. , Piessevaux H. , Horsmans Y. , Gala J. L. , and Belgian Inflammatory Bowel Disease Research Group (BIRD) , Limitations of Extensive TPMT Genotyping in the Management of Azathioprine-Induced Myelosuppression in IBD Patients, Clinical Biochemistry. (2011) 44, no. 13, 1062–1066, 10.1016/j.clinbiochem.2011.06.079, 2-s2.0-80051697170, 21723857. 21723857

[10] Banerjee R. , Ravikanth V. V. , Pal P. , Bale G. , Avanthi U. S. , Goren I. , Girish B. G. , Mitnala S. , and Reddy D. N. , NUDT15 C415T Variant Compared With TPMT Genotyping in Predicting Azathioprine-Induced Leucopenia: Prospective Analysis of 1014 Inflammatory Bowel Disease Patients in India, Alimentary Pharmacology & Therapeutics. (2020) 52, no. 11–12, 1683–1694, 10.1111/apt.16137, 33111378. 33111378

[11] Eskazan T. , Bakkaloglu O. K. , Toruner M. , Kani H. T. , Cavus B. , Yilmaz V. , Unal N. G. , Atug O. , Cagcag B. , Dogruel M. , Yilmaz E. , Akyuz F. , Erzin Y. Z. , Hatemi A. I. , and Celik A. F. , Comparison of Azathioprine-Induced Pancreatitis and Gastrointestinal Intolerance in IBD: Role of Demographics, Clinical Variables, and HLA DQA1/DRB1 Alleles, Journal of Clinical Medicine. (2025) 14, no. 23, 10.3390/jcm14238539, 41375842. PMC12692811 41375842

[12] Balarajah S. , Martinez-Gili L. , Alexander J. L. , Mullish B. H. , Perry R. W. , Li J. V. , Marchesi J. R. , Parkes M. , Orchard T. R. , Hicks L. C. , Williams H. R. T. , and UK IBD BioResource Investigators , A Large-Scale Comparison of Clinical Outcomes to IBD Therapies in White and South Asian Ethnicities, EClinicalMedicine. (2025) 90, 103644, 10.1016/j.eclinm.2025.103644, 41497517. 41497517 PMC12766419

[13] Park M. S. , Kim D. H. , Kim D. H. , Park S. J. , Hong S. P. , Kim T. I. , Kim W. H. , and Cheon J. H. , Leukopenia Predicts Remission in Patients With Inflammatory Bowel Disease and Behcet′s Disease on Thiopurine Maintenance, Digestive Diseases and Sciences. (2015) 60, no. 1, 195–204, 10.1007/s10620-014-3355-4, 2-s2.0-84922004297, 25239495. 25239495

[14] Disanti W. , Rajapakse R. O. , Korelitz B. I. , Panagopoulos G. , and Bratcher J. , Incidence of Neoplasms in Patients Who Develop Sustained Leukopenia During or After Treatment With 6-Mercaptopurine for Inflammatory Bowel Disease, Clinical Gastroenterology and Hepatology. (2006) 4, no. 8, 1025–1029, 10.1016/j.cgh.2006.03.018, 2-s2.0-33746370700. 16765651

[15] dos Santos R. C. F. , Catapani W. R. , Takahashi A. A. R. , and Waisberg J. , C-Reactive Protein Levels and Prevalence of Leukopenia in Patients with Inflammatory Bowel Disease Treated with Azathioprine and/or Mesalazine: A Real-Life Study, Einstein. (2022) 20, eAO6500, 10.31744/einstein_journal/2022AO6500, 35584442. 35584442 PMC9060644

[16] Singh A. , Mahajan R. , Kedia S. , Dutta A. K. , Anand A. , Bernstein C. N. , Desai D. , Pai C. G. , Makharia G. , Tevethia H. V. , Mak J. W. Y. , Kaur K. , Peddi K. , Ranjan M. K. , Arkkila P. , Kochhar R. , Banerjee R. , Sinha S. K. , Ng S. C. , Hanauer S. , Verma S. , Dutta U. , Midha V. , Mehta V. , Ahuja V. , and Sood A. , Use of thiopurines in Inflammatory Bowel Disease: An Update, Intestinal Research. (2022) 20, no. 1, 11–30, 10.5217/ir.2020.00155, 33845546. 33845546 PMC8831775

[17] de Boer N. K. , Ahuja V. , Almer S. , Ansari A. , Banerjee R. , Barclay M. L. , Begun J. , van Bodegraven A. A. , Colombel J. F. , Epstein D. P. , Florin T. H. , Gearry R. B. , Gisbert J. P. , Leong R. W. , Mulder C. J. J. , Neurath M. F. , Radford-Smith G. L. , Seinen M. L. , Simsek M. , and Sparrow M. P. , Thiopurine Therapy in Inflammatory Bowel Diseases: Making New Friends Should Not Mean Losing Old Ones, Gastroenterology. (2019) 156, no. 1, 11–14, 10.1053/j.gastro.2018.11.039, 2-s2.0-85058876383, 30472233. 30472233

[18] Chaparro M. , Ordás I. , Cabré E. , Garcia-Sanchez V. , Bastida G. , Peñalva M. , Gomollón F. , García-Planella E. , Merino O. , Gutiérrez A. , Esteve M. , Márquez L. , Garcia-Sepulcre M. , Hinojosa J. , Vera I. , Muñoz F. , Mendoza J. L. , Cabriada J. L. , Montoro M. A. , Barreiro-de Acosta M. , Ceña G. , Saro C. , Aldeguer X. , Barrio J. , Maté J. , and Gisbert J. P. , Safety of Thiopurine Therapy in Inflammatory Bowel Disease, Inflammatory Bowel Diseases. (2013) 19, no. 7, 1404–1410, 10.1097/MIB.0b013e318281f28f, 2-s2.0-84879102390. 23665964

[19] Yewale R. V. , Ramakrishna B. S. , Doraisamy B. V. , Basumani P. , Venkataraman J. , Jayaraman K. , Murali A. , Premkumar K. , and Kumar A. S. , Long-Term Safety and Effectiveness of Azathioprine in the Management of Inflammatory Bowel Disease: A Real-World Experience, JGH Open. (2023) 7, no. 9, 599–609, 10.1002/jgh3.12955, 37744710. 37744710 PMC10517446

[20] Grau G. , Brunet-Mas E. , Llovet L. P. , Pedregal P. , Villoria A. , Melcarne L. , Puy A. , Garcia-Sague B. , Frisancho L. E. , Ramírez-Lázaro M. J. , Lario S. , and Calvet X. , Incidence of Myelotoxicity and Other Adverse Effects Related to Thiopurine Starting in Patients With Inflammatory Bowel Disease: Retrospective Observational Study in a Third-Level Hospital, Journal of Clinical Medicine. (2023) 12, no. 20, 10.3390/jcm12206571, 37892708. PMC10607915 37892708

[21] Gisbert J. P. and Gomollón F. , Thiopurine-Induced Myelotoxicity in Patients With Inflammatory Bowel Disease: A Review, Official journal of the American College of Gastroenterology. (2008) 103, no. 7, 1783–1800, 10.1111/j.1572-0241.2008.01848.x, 2-s2.0-50649110056, 18557712. 18557712

[22] Jim R. E. , Maroute C. , Lahlali M. , Séjai A. L. , Abid H. , Lahmidani N. , Mekkaoui A. E. , Mounia E. Y. , Bennajeh D. A. , Ibrahimi S. A. , and Abkari M. E. , Safety Profile of Azathioprine in Inflammatory Bowel Disease, Journal of Gastroenterology. (2025) 15, no. 8, 427–438, 10.4236/ojgas.2025.158039.

[23] Meijer B. , Wilhelm A. J. , Mulder C. J. J. , Bouma G. , van Bodegraven A. A. , and de Boer N. K. H. , Pharmacology of Thiopurine Therapy in Inflammatory Bowel Disease and Complete Blood Cell Count Outcomes: A 5-Year Database Study, Therapeutic Drug Monitoring. (2017) 39, no. 4, 399–405, 10.1097/FTD.0000000000000414, 2-s2.0-85025608604, 28489727. 28489727 PMC5538301

[24] Min D. I. and Monaco A. P. , Complications Associated With Immunosuppressive Therapy and Their Management, Pharmacotherapy: The Journal of Human Pharmacology and Drug Therapy. (1991) 11, no. 5, 119S–125S, 10.1002/j.1875-9114.1991.tb02641.x, 2-s2.0-0026070231, 1745617. 1745617

[25] Hindorf U. , Lindqvist M. , Hildebrand H. , Fagerberg U. , and Almer S. , Adverse Events Leading to Modification of Therapy in a Large Cohort of Patients With Inflammatory Bowel Disease, Alimentary Pharmacology & Therapeutics. (2006) 24, no. 2, 331–342, 10.1111/j.1365-2036.2006.02977.x, 2-s2.0-33745650894, 16842460. 16842460

[26] Lee L. Y. W. , Gardezi A. S. , Santharam V. , Boyd J. , and Lanzon-Miller S. , Effect of Azathioprine Intolerance on Outcomes of Inflammatory Bowel Disease: a Cross-Sectional Study, Frontline Gastroenterology. (2014) 5, no. 1, 40–43, 10.1136/flgastro-2013-100348, 28839749. 28839749 PMC5369706

[27] Gearry R. B. , Barclay M. L. , Burt M. J. , Collett J. A. , and Chapman B. A. , Thiopurine Drug Adverse Effects in a Population of New Zealand Patients With Inflammatory Bowel Disease, Pharmacoepidemiology and Drug Safety. (2004) 13, no. 8, 563–567, 10.1002/pds.926, 2-s2.0-4544239107, 15317038. 15317038

[28] Stournaras E. , Qian W. , Pappas A. , Hong Y. Y. , Shawky R. , Uk Ibd BioResource Investigators , Raine T. , and Parkes M. , Thiopurine Monotherapy Is Effective in Ulcerative Colitis But Significantly Less so in Crohn′s Disease: Long-Term Outcomes for 11 928 Patients in the UK Inflammatory Bowel Disease Bioresource, Gut. (2021) 70, no. 4, 677–686, 10.1136/gutjnl-2019-320185, 33004550. 33004550 PMC7948184

[29] Relling M. V. , Schwab M. , Whirl-Carrillo M. , Suarez-Kurtz G. , Pui C. H. , Stein C. M. , Moyer A. M. , Evans W. E. , Klein T. E. , Antillon-Klussmann F. G. , Caudle K. E. , Kato M. , Yeoh A. E. J. , Schmiegelow K. , and Yang J. J. , Clinical Pharmacogenetics Implementation Consortium Guideline for Thiopurine Dosing Based on TPMT and NUDT15 Genotypes: 2018 Update, Clinical Pharmacology & Therapeutics. (2019) 105, no. 5, 1095–1105, 10.1002/cpt.1304, 2-s2.0-85058034477, 30447069. 30447069 PMC6576267

[30] Ranjan M. K. and Pathak R. , Epidemiology and Demographic Profile of Inflammatory Bowel Disease in Nepal, Journal of Institute of Medicine Nepal. (2023) 45, no. 2, 1–6, 10.59779/jiomnepal.1259.

